# Using decision tree analysis to identify population groups at risk of subjective unmet need for assistance with activities of daily living

**DOI:** 10.1186/s12877-023-04238-w

**Published:** 2023-09-07

**Authors:** Philipp Jaehn, Hella Fügemann, Kathrin Gödde, Christine Holmberg

**Affiliations:** 1 Institute of Social Medicine and Epidemiology, Brandenburg Medical School, Brandenburg an der Havel, Germany; 2 Faculty of Health Sciences Brandenburg, Brandenburg Medical School, Potsdam, Germany; 3 Institute of Public Health, Charité—Universitätsmedizin Berlin, corporate member of Freie Universität Berlin, Humboldt-Universität Zu Berlin, Berlin, Germany

**Keywords:** Conditional inference trees, Social care, Vulnerable groups, Resource allocation, Unmet need for assistance, Activities of daily living

## Abstract

**Background:**

Identifying predictors of subjective unmet need for assistance with activities of daily living (ADL) is necessary to allocate resources in social care effectively to the most vulnerable populations. In this study, we aimed at identifying population groups at risk of subjective unmet need for assistance with ADL and instrumental ADL (IADL) taking complex interaction patterns between multiple predictors into account.

**Methods:**

We included participants aged 55 or older from the cross-sectional German Health Update Study (GEDA 2019/2020-EHIS). Subjective unmet need for assistance was defined as needing any help or more help with ADL (analysis 1) and IADL (analysis 2). Analysis 1 was restricted to participants indicating at least one limitation in ADL (*N* = 1,957). Similarly, analysis 2 was restricted to participants indicating at least one limitation in IADL (*N* = 3,801). Conditional inference trees with a Bonferroni-corrected type 1 error rate were used to build classification models of subjective unmet need for assistance with ADL and IADL, respectively. A total of 36 variables representing sociodemographics and impairments of body function were used as covariates for both analyses. In addition, the area under the receiver operating characteristics curve (AUC) was calculated for each decision tree.

**Results:**

Depressive symptoms according to the PHQ-8 was the most important predictor of subjective unmet need for assistance with ADL. Further classifiers that were selected from the 36 independent variables were gender identity, employment status, severity of pain, marital status, and educational level according to ISCED-11. The AUC of this decision tree was 0.66. Similarly, depressive symptoms was the most important predictor of subjective unmet need for assistance with IADL. In this analysis, further classifiers were severity of pain, social support according to the Oslo-3 scale, self-reported prevalent asthma, and gender identity (AUC = 0.63).

**Conclusions:**

Reporting depressive symptoms was the most important predictor of subjective unmet need for assistance among participants with limitations in ADL or IADL. Our findings do not allow conclusions on causal relationships. Predictive performance of the decision trees should be further investigated before conclusions for practice can be drawn.

**Supplementary Information:**

The online version contains supplementary material available at 10.1186/s12877-023-04238-w.

## Introduction

The demographic change will have a major impact on the population’s health and on health care systems of the European Union (EU). Between 2020 and 2050, the proportion of the population aged 65 or older in the EU-28 countries is expected to increase from 20.4% to 28.5% [1]. In many European countries, the health of the elderly population is impacted by chronic diseases, decline of cognitive and physical functioning, falls, worsening mental health, and multimorbidity [2, 3]. These aspects may severely affect activities of daily living such as personal hygiene or taking medication. Bathing, dressing, using toilets, transferring, continence, and feeding have been defined by Katz et al. as activities of daily living (ADL) to describe limitations in basic functioning of the elderly [4]. More complex tasks were operationalised by Lawton and Brody as instrumental activities of daily living (IADL) which include using the telephone, shopping, food preparation, housekeeping, doing laundry, using transportation, taking medication, and taking care of finances [5]. In the population aged 80 or older in Germany, severe limitations in any ADL were estimated at 13.4% among women and 9.0% among men [6]. In the same age group, severe limitations in IADL were reported to be 35.9% among women and 21.0% among men [6]. Limitations in performing ADL are associated with reduced quality of life, institutionalisation, high health care costs, and increased mortality [7].

As a corollary, providing care and support for people with limitations in ADL and IADL is crucial to strengthen physical, mental and social health of older adults. However, a significant proportion of the German population aged 55 or older with limitations in ADL or IADL report a lack of sufficient support. For example, when experiencing severe limitations in ADL, 47.4% state that more support is needed [6]. For people with severe limitations in IADL, this figure is 28.3%, indicating that informal support structures are better suited to compensate complex tasks in the household compared to basic needs for body care and personal hygiene [6, 8]. Furthermore, several studies show that an unmet need for assistance with ADL or IADL is related to a higher risk of rehospitalisation, psychological distress, mortality, and impaired healthy aging [9–13]. It is important to note that the ADL and IADL scales consider only a part of the spectrum of functioning and disability as defined by the International Classification of Functioning, Disability and Health (ICF). The ICF contains a wide range of activities in the domains “Learning and applying knowledge”, “General tasks and demands”, “Communication”, “Mobility”, “Self care”, “Domestic life”, “Interpersonal interactions and relationships”, “Major life areas”, and “Community, social and civic life” [14]. The ADL scale by Katz et al. covers a confined selection of activities from the domains of mobility and self care, while the IADL scale by Lawton and Brody considers a selection of activities from the domains communication, mobility, and domestic life [4, 5].

Information on predictors of subjective unmet need for assistance is necessary for health care providers to identify underserved population groups. For example, this knowledge may be useful for the development of patient navigation programs. Patient navigators assist in identifying personal health care needs, support with administrative tasks in complex health care systems, provide specific knowledge on health issues, and offer emotional support for people with severely limiting health conditions [15]. In this paper, we categorise predictors of subjective unmet need for assistance with ADL or IADL into socio-demographic factors and impairments. According to the ICF, impairments depict any problems in body function and structure such as significant deviation or loss [14]. To describe the available evidence on associated factors, we focus on studies from western European countries, because we assume that subjective unmet need for assistance is highly context-specific. Sociodemographic factors that were associated with subjective unmet need for assistance in studies from Germany and England were female sex, living alone, belonging to a low occupational social class and having a low level of education [6, 16]. Furthermore, the study from England showed that impairments such as bad subjective health and having a limiting long-term illness are associated with subjective unmet need for assistance [16]. Additional findings are available from research outside Europe. Studies from China, Taiwan, Chile, the United States of America (USA), and South Africa, found that a higher degree of physical disability, a higher degree of cognitive impairment, and a higher number of limitations in ADL or IADL are associated with a higher chance of reporting unmet need for assistance [17–21].

In the contemporary scientific literature, however, it remains unclear if predictors of subjective unmet need for assistance interact in complex patterns. Intersectionality-informed research has suggested that considering complex interactions may yield a more precise picture of lived realities and may aid in identifying underserved population groups more accurately [22]. Decision-tree analysis is a data-driven exploratory approach capable of considering complex interactions among a large number of potential predictors of an outcome [22, 23].

## Methods

### Aim

The aim of this work was to use conditional inference trees to consider complex interactions between a large set of potential predictors for subjective unmet need for assistance with ADL and subjective unmet need for assistance with IADL. Potential predictors were a selection of socio-demographic factors and impairments in body function or structure.

### Study design and population

We used the scientific use file of the cross-sectional study German Health Update (GEDA 2019/2020-EHIS) which has been carried out by the Robert Koch-Institute in 2019 and 2020 [24]. The study was conducted among the population aged 15 or older with a usual residence in a German private single or multi-person household. Care and residential homes were not included in the survey. The survey was based on a telephone sample that has been drawn using the dual-frame method, which considers landline and mobile phone numbers. After obtaining informed consent, Computer Assisted Telephone Interviews (CATI) were applied to collect the data. No proxy interviews have been conducted among people with cognitive or sensory impairment. The “Response Rate 3” of the American Association for Public Opinion Research (AAPOR) classification was 21.6%. Finally, survey weights were calculated by the principal investigators of the study using external information on federal state, residential structure, age, sex, and education to reduce a possible non-response bias. These survey weights were also provided to the authors in the scientific use file. More details about the survey methodology and data quality assurance procedures can be obtained elsewhere [25]. Overall, 23,001 participants were interviewed in GEDA 2019/2020-EHIS. We included the population aged 55 or older (*N* = 12,985, 56.5% of the original sample), because items on ADL and IADL were only applied in this age group. GEDA 2019/2020-EHIS included an ADL score according to Katz et al. and an IADL score according to Lawton and Brody [4, 5]. The entire GEDA 2019/2020-EHIS questionnaire can be found elsewhere [26]. To assess limitations in ADL, five items asking for limitations in feeding, transferring, dressing, using toilets, and bathing were used. Response options to each item were “no difficulties”, “some difficulties”, “severe difficulties”, and “it´s not possible for me”. In the IADL score, seven items asked for limitations in food preparation, using the telephone, shopping, taking medication, light housework, heavy housework, and taking care of finances. For IADL items, response options were “no difficulty”, “some difficulty”, “severe difficulty”, “it is not possible for me”, and “have never tried or done it”. The latter response option was considered missing in the IADL items.

In the analysis of subjective unmet need for assistance with ADL, we only included participants who reported “some difficulty”, “severe difficulty”, or “it is not possible for me” for at least one activity of the ADL score. We excluded all participants who only responded with”no difficulty” in all available ADL items as well as participants who had missing observations in all available ADL items (final *N* = 1,957, 8.5% of the original sample). In the analysis of subjective unmet need for assistance with IADL, we applied similar inclusion and exclusion criteria. Participants indicating “some difficulty”, “severe difficulty”, or “it is not possible for me” for at least one activity of the IADL score were included. Participants responding only”no difficulty” in available IADL items or having missing values in all available IADL items were excluded (final *N* = 3,801, 16.5% of the original sample).

### Variables

The two outcomes of interest in our study were subjective unmet need for assistance with ADL and subjective unmet need for assistance with IADL. In the questionnaire of the GEDA 2019/2020-EHIS study, questions on subjective unmet need for assistance were asked directly after the items of the ADL and IADL scores, respectively. Concerning the first outcome, participants were asked if they had any help with ADL tasks in general (“Do you usually have help with these activities? Now think about activities related to personal hygiene and other personal needs that you have difficulty performing without help.”). Response options were “Yes, for at least one activity” and “No”. After this question, all participants who stated that they had no help were asked if they needed any help (“Do you need help?”). All participants who answered that they had help were asked if they needed more help (“Do you need more help?”). Both questions could be answered with “yes” and “no”. In our study, all individuals who stated that they needed any help or that they needed more help were classified as having subjective unmet need for assistance with ADL. Subjective unmet need for assistance with IADL was assessed in the same manner. Only the phrasing of the initial question for subjective unmet need for assistance with IADL tasks differed from the ADL section (“Do you usually have help with these activities? Now think of all the activities in the household that you have difficulty doing without help.”).

We included 14 potential sociodemographic predictors of subjective unmet need for assistance: gender identity (female, male, other) [27], 5-year age group, migrant status (two-sided migration background, no or one-sided migration background), rurality of the district of residence (large city, urban district, rural district, sparsely populated rural district), educational level according to the International Standard Classification of Education (ISCED, levels 1–8), household income (quintiles), employment status (employed, unemployed, retired, unable to work due to long-term illness, doing housework, not working for other reasons), main earner in the household (myself, there is no main breadwinner, my partner), marital status (single, married, widowed, divorced), living with partner (yes, no), number of people in the household (1, 2, 3, 4, 5, 6, 7 or more), health insurance type (statutory health insurance, private insurance, other), Oslo-3 scale for social support (low, medium, high) [28], time spent caring for others (none, less than 10 h per week, at least 10 but less than 20 h per week, 20 h per week or more).

Twenty two variables were used to mirror a selection of impairments in body function or structure. We used single questions with the response options yes and no to measure the following self-reported diseases that were prevalent in the last 12 months: hypertension, myocardial infarction, coronary artery disease, stroke, diabetes, asthma, hypolipoproteinaemia, chronic bronchitis, liver cirrhosis, chronic kidney disease, urinary incontinence, any allergy, arthrosis, complaints of neck or cervical spine, complaints of lower back, injury due to traffic accident, injury due to home accident, injury due to accident in free time. Furthermore, the Patient Health Questionnaire 8 (PHQ-8) [29] score for depressive symptoms in the past 2 weeks was included alongside the severity of pain in the last 4 weeks (no pain, very mild, mild, moderate, severe, very severe). The total score of the PHQ-8 is determined by summing up four single items (each on a scale 0–3). The total PHQ-8 score ranges from 0 to 12 and represents a metric on the ordinal scale [29]. Moreover, visual difficulty (none without visual aid, none with visual aid, difficulties with visual aid, difficulties without visual aid) and hearing difficulty (none, moderate, severe) were considered.

### Statistical methods

We used conditional inference trees to select predictors of subjective unmet need for assistance with ADL and IADL tasks, respectively. Conditional inference trees use a procedure based on a regression coefficient and a hypothesis test of a global null hypothesis. The global null hypothesis is the hypothesis that none of the covariates has a univariate association with the outcome. If the algorithm selects a first split of the data, it assesses subsequent splits in the resulting two subsets of the data. This process represents a decision algorithm, which produces a sequence of hierarchical binary decisions that can be graphically displayed as a tree. To deal with multiple testing, an overall Bonferroni-corrected type on error rate (alpha) is chosen in conditional inference trees and serves as stopping criterion in the tree-building process. Generated subsets of the data are called nodes and final subsets represent terminal nodes. Each observation can be allocated to a single terminal node [23, 30].

Missing observations in the two outcomes were not included in our analyses. The first outcome, subjective unmet need for assistance with ADL, had 8 (0.4%) missing observations while the variable subjective unmet need for assistance with IADL had 22 (0.6%) missings. Our selection of 14 socio-demographic variables and 22 variables indicating impairments of body function were used together as independent variables in the analyses of both outcomes. Missing observations in independent variables were not excluded. It is possible to maintain missing values of independent variables in conditional inference tree analyses since the algorithm uses so-called surrogate splits to deal with missing observations [23]. Survey weights that were provided in the scientific use file were applied in both decision trees to correct for non-response. The minimum node size in a terminal node was restricted to 1% of the weight of the overall population. Trees were grown using the package partykit (version 1.2–15) in R (version 4.0.2) [31].

Finally, the area under the receiver operating characteristics curve (AUC) was calculated for each tree to assess discriminatory accuracy. The AUC combines the true positive and the false positive rate in a single metric for discriminatory accuracy of a binary outcome. The metric has a range from 0.5 to 1 where 1 represents perfect discrimination [32]. In our case, the AUC shows the ability of a decision tree to predict unmet need for assistance. Finally, trees were very large when using alpha = 5% which impacted interpretability of our results. Hence, we used cross-validation (CV), to evaluate if there are values for alpha that lead to smaller trees with no or little loss of discriminatory accuracy as measured by the AUC [33]. Tenfold CV showed that alpha = 0.5% did not result in a substantive loss of AUC in the analysis of subjective unmet need for assistance with ADL. For the analysis of subjective unmet need for assistance with IADL, alpha = 0.1% was chosen based on CV. The impact of this choice was shown by calculating AUC for all trees with alpha = 5% and for the lower levels of alpha that were chosen based on CV.

## Results

### Description of sample characteristics

Among participants who reported difficulty with at least one ADL (*N* = 1,957), 22.6% reported a need for support with ADL, 41.1% reported a male gender identity and 0.4% reported an identity that was not female or male (Table 1). 73.8% were over 65 years. Furthermore, 6.9% had a two-sided migration background, 11.9% lived in a sparsely populated rural district, and 23.4% had low social support according to the Oslo-3 scale. Moreover, 6.8% experienced a stroke in the past 12 months. Among participants who reported a difficulty in IADL (*N* = 3,801), 22.8% reported a need for support with IADL, 35.2% reported a male gender identity, and 0.4% reported an identity that was not female or male. 72.2% were over 65 years of age, 7.0% had a two-sided migration background, 11.0% lived in a sparsely populated rural district, and 20.1% reported low social support. Finally, 5.3% had a stroke in the past 12 months. All included independent variables are summarised in the additional file (Additional file 1).

**Table 1:** Description of the samples used in analyses of unmet need for assistance in tasks of (instrumental) activities of daily living

	ADL	Need for more assistance (ADL)	IADL	Need for more assistance (IADL)
		(*N* = 443, 22.6%)		(*N* = 1057, 27.8%)
	N	%	N	%
**Gender identity**				
male	804	20.0	1337	24.6
female	1137	24.7	2447	29.7
other	8	12.5	17	37.5
**Age group**				
55–59	242	21.9	522	29.4
60–64	263	14.4	533	28.2
65–69	252	19.4	552	24.0
70–74	235	22.1	475	28.3
75–79	347	23.3	680	26.8
80–84	353	27.8	631	29.9
85–89	183	26.8	295	29.1
over 90	74	23.1	113	31.9
**Migrant status**				
two-sided	136	27.7	266	30.6
none or one-sided	1800	22.3	3508	27.7
missing	13 (0.7%)		27 (0.7%)	
**Rurality of district of residence**				
large city	679	21.1	1268	27.0
urban district	657	23.3	1405	27.6
rural district	245	21.6	449	32.8
sparsely populated rural district	232	22.4	417	24.0
Missing	136 (7.0%)		262 (6.9%)	
**Household income (quintiles)**				
1 (low)	370	27.3	660	32.0
2	431	24.6	824	30.8
3	397	24.2	752	29.1
4	395	19.2	790	25.8
5 (high)	334	17.7	722	21.2
Missing	22 (1.1%)		53 (1.4%)	
**Social support (Oslo-3)**				
low	457	33.5	763	37.6
medium	865	17.9	1717	26.2
high	523	20.5	1140	24.4
Missing	104 (5.3%)		181 (4.8%)	

No adjustment weights were applied in the calculations of numbers of observations and proportions

*ADL* Activities of daily living, *IADL* Instrumental activities of daily living

### Results for participants reporting difficulties in ADL

**Fig. 1:**
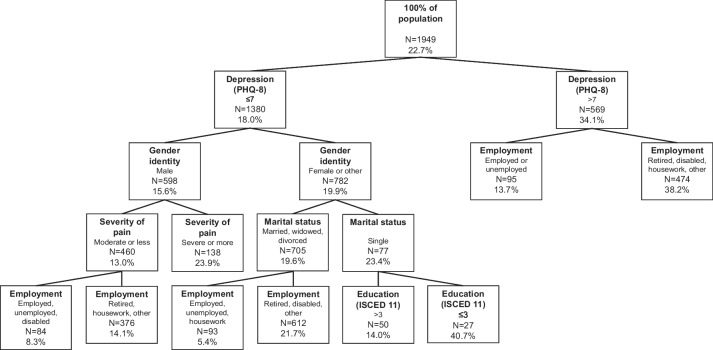
Decision tree for the identification of population groups at risk of subjective unmet need for assistance with ADL. Legend: N are numbers of observations in the corresponding node of the tree. Percentages are proportions of participants with unmet need for assistance in the corresponding node of the tree. PHQ-8: Patient Health Questionnaire 8 ISCED 11: International Standard Classification of Education 11

In the analysis of predictors for subjective unmet need for assistance with ADL, the first split occurred for depressive symptoms (PHQ-8 higher seven) (Fig. 1). Among participants with a PHQ-8 lower or equal to seven, the proportion of subjective unmet need for assistance was 18.0%, while it was 34.1% for participants who showed a PHQ-8 higher seven. A subsequent split occurred according to gender identity in the subset of participants with a PHQ-8 lower or equal to seven. The proportion of subjective unmet need for assistance was 19.9% among people with female or other gender identity compared to 15.6% among people identifying as male in this subset. In the subset with male gender identity, the next split was induced for severity of pain, with a proportion of subjective unmet need for assistance of 23.9% among the subset with a severe or very severe intensity of pain. On the other hand, in the subset with moderate or less severe intensity of pain, 13.0% reported an unmet need for assistance. In this node, a final split occurred for employment status. Turning back to the node of people with a PHQ-8 lower or equal to seven and a female or other gender identity, the next split was induced for marital status. Single people had a higher proportion of subjective unmet need for assistance (23.2%) compared to people with another marital status (19.6%) in this subset. In this node with a non-single marital status, the final split was induced for employment status. In the node with a marital status “single”, the final split occurred for level of education. Finally, just one split occurred on the other half of the tree. The node with a PHQ-8 higher than seven was split according to employment status. Subjective unmet need for assistance was lower in the subset who were employed or unemployed (13.7%) compared to the subset in retirement or in another form of employment (38.2%). The AUC of the decision tree was 0.66 when using alpha = 0.5% and 0.66 when using alpha = 5%.

### Results for participants reporting difficulties in IADL

**Fig. 2:**
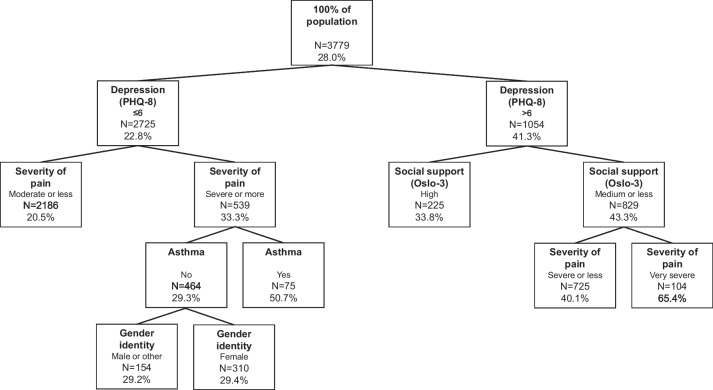
Decision tree for the identification of population groups at risk of subjective unmet need for assistance with IADL. Legend: N are numbers of observations in the corresponding node of the tree. Percentages are proportions of participants with unmet need for assistance in the corresponding node of the tree. PHQ-8: Patient Health Questionnaire 8 Oslo-3: Oslo social support scale 3

Among participants reporting difficulties in IADL, depressive symptoms also induced the first split (PHQ-8 higher six) (Fig. 2). The proportion of subjective unmet need for assistance was 22.8% among participants with a PHQ-8 score lower or equal to six compared to 41.3% among participants with a score higher than six. The former node (PHQ-8 score lower or equal to six) was split further according to severity of pain. The subset with a moderate or less severe intensity of pain showed a lower proportion of subjective unmet need for assistance (20.5%) compared to the subset with severe or very severe intensity (33.3%). In the latter node, the next split was induced for prevalent self-reported asthma. In the subset of participants reporting an asthma diagnosis in the past 12 months, 50.7% reported having an unmet need for assistance. In the subset with no self-reported asthma diagnosis, 29.3% had a subjective unmet need for assistance. Finally, a last split occurred in the subgroup with no asthma diagnosis according to gender identity with a higher proportion of subjective unmet need for assistance among people with female identity (29.4%) compared to people with male or other identity (29.2%). On the other half of the tree (PHQ-8 score higher than six), the subsequent split after depressive symptoms occurred for social support. In the node with high social support according to Oslo-3, the proportion of subjective unmet need for assistance was 33.8%. In contrast, 43.3% reported a subjective unmet need for assistance in the node with medium or less social support. A final split occurred in this subset. The terminal node with severe or less intense pain showed a lower proportion of subjective unmet need for assistance (40.1%) compared to the terminal node with very severe pain (65.4%). The AUC in the final tree for the prediction of subjective unmet need for assistance in IADL was 0.63 when using alpha = 0.1% and 0.64 when using alpha = 5%.

## Discussion

In this study, we found that depressive symptoms was the most important characteristic for the identification of population groups with comparatively high unmet need for assistance with both ADL and IADL. For ADL, gender identity and employment status were further important classifiers. In contrast, severity of pain and social support according to Oslo-3 were subsequent important predictors of subjective unmet need for assistance with IADL. The decision tree for the prediction of unmet need for assistance with ADL yielded an AUC of 0.66 compared to an AUC of 0.63 for the prediction of unmet need for assistance with IADL.

This study had several limitations. First, we used cross-sectional data. Longitudinal data would be more desirable to develop prediction models and to assess their discriminatory accuracy. The self-reported nature of the data presents a further limitation, possibly impacting on measurement accuracy. Concerning measurement, it is important to mention, that the items of the ADL and IADL score do not comprehensively capture the ICF domains which comprise a large selection of characteristics from the domains of mobility, self care, communication, mobility, and domestic life. The ADL and IADL scores used in our study mirror a limited selection of these activities. Furthermore, we used a binary variable for subjective unmet need for assistance and future studies should attempt to distinguish different levels of the extent of help that is needed. This would mean to apply statistical models that are able to incorporate dependent variables beyond the binary. Moreover, it is important to note that this study did not include people living in care homes. Hence, results are not generalizable to this population group. Finally, our decision trees should be validated in further study populations before a final conclusion on the predictive capacity of the model can be made. A strength of this study is the large number of potential predictors that could be included. Using data of the GEDA 2019/2020-EHIS survey, we were able assess 36 independent variables. Intentionally, we only included variables that indicated specific diseases rather than variables on general health in order to provide results that are more precise. Furthermore, the sample size of the study was large, enabling to identify small population subgroups with comparably high subjective unmet need for assistance. Finally, the method of conditional inference trees was a data-driven approach, possibly reducing subjective bias by the analyst when selecting variables and enabling to consider interactions between multiple independent variables.

Compared to studies from Germany and England, we found similar variables to be predictors of subjective unmet need for assistance with ADL or IADL. A previous study from Germany that used the same dataset found that female sex assigned at birth was associated with a high unmet need for help with ADL but not with IADL tasks [6]. In addition, our results agree with a study from the United Kingdom. However, this study applied an “absolute” approach to operationalise unmet need for care, where only absence of any help was considered as unmet need [16]. As a corollary, the results are only comparable to a limited extent. In the univariable analysis, old age was associated with higher unmet need with ADL and IADL [16]. In our study, age was not selected by the decision tree algorithm, however, retirement may reflect the relationship with old age. In contrast to our study, sex was not associated with either ADL or IADL tasks [16]. Comparable to our findings, international studies have also found that factors related to loneliness and missing social networks are important predictors of subjective unmet need for assistance [19, 21, 34]. Finally, our study is in line with previous results showing that a low educational level is associated with unmet need for assistance [19, 20].

Considering impairments of body function or structure, our analysis is comparable to a previous study that highlighted the importance of mental health for unmet need for assistance [35]. In both of our assessed outcomes, depression according to PHQ-8 was the most important predictor. However, the importance of mental health seems to receive little attention in the current literature overall. In addition, our study highlights the importance of the subjectively experienced pain severity. We found no study that investigated the association of pain with unmet need for assistance. We would interpret this finding as another advantage of a decision tree analysis, because this predictor was not considered in past theorising and conceptualisations of unmet need for care and was not identified in past empirical studies.

Our study should be interpreted as a prediction exercise to identify subgroups at risk of subjective unmet need for assistance. The advantage of a decision tree analysis is the data-driven selection of classifiers to discriminate presence and absence of subjective unmet need for assistance. In this process, a large number of variables is reduced to the most important predictors, which may be easy to convey to policy-makers. The graphical display of the results may support an intuitive presentation of results. However, factors with lower strengths of associations may not be chosen by the algorithm despite being true causes of the outcome. In contrast to our study, a causal model of unmet need for assistance should consider a wide variety of factors related to the socio-political context next to variables in the individual level. These are conditions such as housing arrangements [36], access to professional services [37], funding for social care [38], and the broader policy context [39–41]. Moreover, our results represent contrasts between averages that were chosen based on multiple hypothesis tests. Hence, the analysis cannot capture the relevance of need for assistance for the individual. Finally, the AUC values of 0.66 and 0.63 show that much inter-individual heterogeneity remains unexplained within the subgroups chosen by the decision-tree algorithm. These AUC values do not represent a good predictive performance [32]. Hence, more research is needed to find predictors that can distinguish populations with and without subjective unmet need for assistance more precisely.

## Conclusion

In conclusion, we have shown that decision trees yield a selection of important predictors of subjective unmet need for assistance with ADL and IADL. Our analysis points towards the importance of mental health, namely depressive symptoms. The analysis approach was able to take complex interactions of a wide variety of potential predictors into account, which resulted in a sequence of decisions that may not have been identified in traditional analysis techniques such as regression. Moreover, pain was identified as an important classifier, which was overlooked in past research. We hope that further studies use this approach to build prediction models in prospective studies. However, predictive performance of the decision trees was poor and further research is needed to identify more precise prediction models.

### Supplementary Information

**Additional file 1: **Descriptive statistics of included independent variables. **Table S1.** Numbers of observations and proportions of sociodemographic characteristics (unweighted). **Table S2.** Numbers of observations and proportions of impairments of body function or structure (unweighted).

## Data Availability

The datasets used and/or analysed during the current study are available from the Robert Koch-Institute on reasonable request at https://www.rki.de/EN/Content/Health_Monitoring/Public_Use_Files/public_use_file_node.html. Dataset used: Robert Koch-Institute, Department of Epidemiology and Health Monitoring (2022): German Health Update 2019/2020-EHIS (GEDA 2019/2020-EHIS). Scientific Use first version. Version. https://doi.org/10.7797/31-201920-1-1-1.

## Abbreviations

ADL: Activities of Daily Living
AUC: Area under the receiver operating characteristics curve
CATI: Computer Assisted Telephone Interview
CV: Cross-validation
GEDA: German Health Update
IADL: Instrumental Activities of Daily Living
ISCED: International Standard Classification of Education
PHQ-8: Patient Health Questionnaire 8
UK: United Kingdom
USA: United States of America
