# Supplementary material for: Using decision tree analysis to identify population groups at risk of subjective unmet need for assistance with activities of daily living
Source: BMC Geriatr. 2023 Sep 7;23:543. doi: 10.1186/s12877-023-04238-w (PMC10483760; doi:10.1186/s12877-023-04238-w)
Supplement: Supplementary file 1 — Additional file 1: Descriptive statistics of included independent variables. Table S1. Numbers of observations and proportions of sociodemographic characteristics (unweighted). Table S2. Numbers of observations and proportions of impairments of body function or structure (unweighted). [file 12877_2023_4238_MOESM1_ESM.pdf]

Additional file 1: Descriptive statistics of included independent variables

Table 1: Numbers of observations and proportions of sociodemographic characteristics (unweighted)

	ADL		IADL	
	N	%	N	%
**Gender identity**				
male	804	41.3	1337	35.2
female	1137	58.3	2447	64.4
other	8	0.4	17	0.4
**Age group**				
55-59	242	12.4	522	13.7
60-64	263	13.5	533	14.0
65-69	252	12.9	552	14.5
70-74	235	12.1	475	12.5
75-79	347	17.8	680	17.9
80-84	353	18.1	631	16.6
85-89	183	9.4	295	7.8
over 90	74	3.8	113	3.0
**Migrant status**				
two-sided	136	7.0	266	7.0
none or one-sided	1800	92.4	3508	92.3
missing	13	0.7	27	0.7
**Rurality of district of residence**				
large city	679	34.8	1268	33.4
urban district	657	33.7	1405	37.0
rural district	245	12.6	449	11.8
sparsely populated rural district	232	11.9	417	11.0
missing	136	7.0	262	6.9
**Education (ISCED 11)**				
1	10	0.5	13	0.3
2	206	10.6	360	9.5
3	802	41.1	1533	40.3
4	109	5.6	256	6.7
6	535	27.4	1041	27.4
7	228	11.7	491	12.9
8	50	2.6	94	2.5
missing	9	0.5	13	0.3
**Household income (quintiles)**				
1 (low)	370	19.0	660	17.4
2	431	22.1	824	21.7
3	397	20.4	752	19.8
4	395	20.3	790	20.8
5 (high)	334	17.1	722	19.0
missing	22	1.1	53	1.4

ADL: activities of daily living, IADL: instrumental activities of daily living, ISCED: International Standard Classification of Education

Table 1: (continued)

	ADL		IADL	
	N	%	N	%
**Employment**				
employed	257	13.2	600	15.8
unemployed	32	1.6	70	1.8
retired	1487	76.3	2797	73.6
permanently disabled	121	6.2	211	5.6
doing housework	26	1.3	66	1.7
not working for other reasons	26	1.3	54	1.4
missing	0	0.0	3	0.1
**Main earner in the household**				
myself	1369	70.2	2518	66.2
there is no main breadwinner	281	14.4	546	14.4
my partner	287	14.7	712	18.7
missing	12	0.6	25	0.7
**Marital status**				
single	201	10.3	370	9.7
married	856	43.9	1804	47.5
widowed	626	32.1	1131	29.8
divorced	265	13.6	492	12.9
missing	1	0.1	4	0.1
**Living with partner**				
yes	852	43.7	1811	47.6
no	1091	56.0	1975	52.0
missing	6	0.3	15	0.4
**People in household**				
1	1011	51.9	1831	48.2
2	826	42.4	1711	45.0
3	74	3.8	180	4.7
4	22	1.1	41	1.1
5	9	0.5	21	0.6
6	3	0.2	8	0.2
7 or more	2	0.1	4	0.1
missing	2	0.1	5	0.1
**Health insurance type**				
statutory health insurance	1676	86.0	3219	84.7
private health insurance	258	13.2	556	14.6
other	11	0.6	21	0.6
missing	4	0.2	5	0.1
**Social support (Oslo-3)**				
low	457	23.4	763	20.1
medium	865	44.4	1717	45.2
high	523	26.8	1140	30.0
missing	104	5.3	181	4.8

ADL: activities of daily living, IADL: instrumental activities of daily living

Table 1: (continued)

	ADL		IADL	
	N	%	N	%
**Time spent caring for others**				
none	1542	79.1	2933	77.2
less than 10 hours per week	242	12.4	549	14.4
min. 10 but max. 20 hours per week	87	4.5	168	4.4
20 hours per week or more	74	3.8	148	3.9
missing	4	0.2	3	0.1

ADL: activities of daily living, IADL: instrumental activities of daily living

Table 2: Numbers of observations and proportions of impairments of body function or structure (unweighted)

	ADL		IADL	
	N	%	N	%
**Hypertension**				
yes	1183	60.7	2138	56.2
no	755	38.7	1642	43.2
missing	11	0.6	21	0.6
**Myocardial infarction**				
yes	98	5.0	165	4.3
no	1850	94.9	3635	95.6
missing	1	0.1	1	0.0
**Coronary artery disease**				
yes	338	17.3	545	14.3
no	1568	80.5	3187	83.8
missing	43	2.2	69	1.8
**Stroke**				
yes	134	6.9	201	5.3
no	1814	93.1	3597	94.6
missing	1	0.1	3	0.1
**Diabetes**				
yes	473	24.3	795	20.9
no	1469	75.4	2997	78.8
missing	7	0.4	9	0.2
**Asthma**				
yes	226	11.6	442	11.6
no	1714	87.9	3350	88.1
missing	9	0.5	9	0.2
**Hypolipoproteinaemia**				
yes	774	39.7	1424	37.5
no	1100	56.4	2247	59.1
missing	75	3.8	130	3.4
**Chronic bronchitis**				
yes	316	16.2	568	14.9
no	1623	83.3	3215	84.6
missing	10	0.5	18	0.5
**Liver cirrhosis**				
yes	30	1.5	43	1.1
no	1907	97.8	3740	98.4
missing	12	0.6	18	0.5
**Chronic kidney disease**				
yes	168	8.6	259	6.8
no	1769	90.8	3529	92.8
missing	12	0.6	13	0.3

ADL: activities of daily living, IADL: instrumental activities of daily living

Table 2: (continued)

	ADL		IADL	
	N	%	N	%
**Urinary incontinence**				
yes	617	31.7	927	24.4
no	1325	68.0	2865	75.4
missing	7	0.4	9	0.2
**Any allergy**				
yes	562	28.8	1120	29.5
no	1377	70.7	2664	70.1
missing	10	0.5	17	0.4
**Arthrosis**				
yes	1034	53.1	1722	45.3
no	877	45.0	2020	53.1
missing	38	1.9	59	1.6
**Complaints of neck or cervical spine**				
yes	1291	66.2	2254	59.3
no	658	33.8	1536	40.4
missing	8	0.4	11	0.3
**Complaints of lower back**				
yes	859	44.1	1543	40.6
no	1092	56.0	2246	59.1
missing	6	0.3	12	0.3
**Injury due to traffic accident**				
yes	33	1.7	60	1.6
no	1914	98.2	3739	98.4
missing	2	0.1	2	0.1
**Injury due to home accident**				
yes	235	12.1	358	9.4
no	1711	87.8	3440	90.5
missing	3	0.2	3	0.1
**Injury due to accident in free time**				
yes	140	7.2	216	5.7
no	1807	92.7	3583	94.3
missing	2	0.1	2	0.1
**Depression (PHQ-8)**				
Continuous score	Mean (SD): 5.85 (4.56)		Mean (SD): 5.05 (4.26)	
missing	99 (5.1%)		160 (4.2%)	
**Severity of pain**				
no pain	198	10.2	669	17.6
very mild	84	4.3	225	5.9
mild	367	18.8	833	21.9
moderate	577	29.6	1047	27.5
severe	524	26.9	771	20.3
very severe	193	9.9	246	6.5
missing	6	0.3	10	0.3

ADL: activities of daily living, IADL: instrumental activities of daily living, PHQ-8: Patient Health Questionnaire 8

Table 2: (continued)

	ADL		IADL	
	N	%	N	%
**Visual difficulty**				
none with visual aid	1075	55.2	2270	59.7
difficulties with visual aid	583	29.9	1024	26.9
difficulties without visual aid	107	5.5	171	4.5
none without visual aid	115	5.9	227	6.0
missing	69	3.5	109	2.9
**Hearing difficulty**				
none	833	42.7	1714	45.1
moderate	844	43.3	1663	43.8
severe	272	14.0	422	11.1
missing	0	0.0	2	0.1

ADL: activities of daily living, IADL: instrumental activities of daily living
